# Supplementary material for: Novel diamond shuttle to deliver flexible neural probe with reduced tissue compression
Source: Microsyst Nanoeng. 2020 Jun 1;6:37. doi: 10.1038/s41378-020-0149-z (PMC7261651; doi:10.1038/s41378-020-0149-z)
Supplement: Supplementary file 1 — Supplemental Materials [file 41378_2020_149_MOESM1_ESM.docx]

**Supplemental Notes**

**Novel diamond shuttle to deliver flexible bioelectronics with reduced tissue compression**

Kyounghwan Na^1^, Zachariah J. Sperry^2,3^, Jiaao Lu^1^, Mihaly Vöröslakos^1,4^, Saman S. Parizi^1^, Tim M. Bruns^2,3^, Euisik Yoon^1,5,6^, John P. Seymour^1,7,8^

Affiliations

^1^Electrical Engineering & Computer Science Department, University of Michigan, Ann Arbor, MI 48105, USA

^2^Biomedical Engineering Department, University of Michigan, Ann Arbor, MI 48105, USA

^3^Biointerfaces Institute, University of Michigan, Ann Arbor, MI 48105, USA

^4^The Neuroscience Institute, New York University, New York, NY 10016, USA

^5^Center for Nanomedicine, Institute for Basic Science (IBS), Seoul 03722, Korea

^6^Graduate Program of Nano Biomedical Engineering (Nano BME), Yonsei-IBS Institute

Yonsei University, Seoul 03722, Korea

^7^University of Texas Health Science Center, Houston, TX 77030, USA

^8^Rice University, Houston, TX 77005, USA

## Table of Contents:

Supplemental Note 1: Mounting polymer arrays to the shuttle

Supplementary Figure 1: Simulation of blood vessel invasion based on various probe cross-sections

Supplemental Note 2: Limits to conformal trench filling with UNCD

Supplemental Figure 2: Simulation result of UNCD profile modeling

Supplementary Figure 3: Measurement setup and force-distance curve for insertion through phantom

Supplementary Figure 4: Phantom insertion force of 1,2,4 and 24 shanks for UNCD

Supplementary Figure 5: Maximum compression distance of UNCD shuttle with varying oscillation frequency at 2 mm/s in tissue phantom

Supplementary Figure 6: Kinematics of instantaneous insertion speed using a piezostage motor with uniaxial motion

Supplementary Table 1: Peak velocity and acceleration of all phantom conditions tested

Supplementary Figure 7: Oscillation and force testing of a 2-µm tip pipette at low speeds in tissue phantom

Supplementary Figure 8: Picture and impedance of PEDOT:pTS coated electrodes

Supplementary Note 3: Detailed results of feline in-vivo experiments

Supplementary Figure 9: Recorded neural signal from somatosensory cortex of rat

Supplementary Note 4: Calculation of moment of inertia of trapezoid shaped T-beam

Supplementary Video 1: Side-by-side comparison of UNCD insertion without and with 200 Hz oscillation.

Supplementary Video 2: In vivo insertion through rat dura and retraction leaving flexible array implanted

Supplementary Video 3: Example of insertion failure due to tissue movement during ex-vivo trial.

References

## Supplemental Note 1: Mounting polymer arrays to the shuttle

Regarding the temporary adhesion of a polymer array onto the diamond shuttle, we describe our PEG mounting process below. The dissolution rate of PEG has to be carefully controlled so that the array doesn’t separate prematurely during insertion and the dissolution and separation happens within a reasonable time after insertion. For cat DRG, this is not trivial because there are several hundred microns of relative DRG position shifting as the vertebral region is coupled to the body’s movement during breathing. This movement is enough to either cause damage to the tissue or fracture the shuttle. We found that if the UNCD shuttle is removed within five minutes of insertion it did not break in three of three attempts. When left for longer, the shuttle fractured. Comparatively, a head-fixed rat surgery had no time limit and movement was hardly visible (Supplementary Video 3), although Gilletti et. al found this to be around 10-30 µm and varied with anesthesia^1^. We tested the molecular weight of PEG (4,000, 8,000, and 12,000 MW) and found the best result for our purposes to be dip coating with 12,000 MW. For rapid removal, using the T-UNCD as a micropost is expected to be as effective as other microposts^2,3^ except it would also be stiffer and/or smaller.


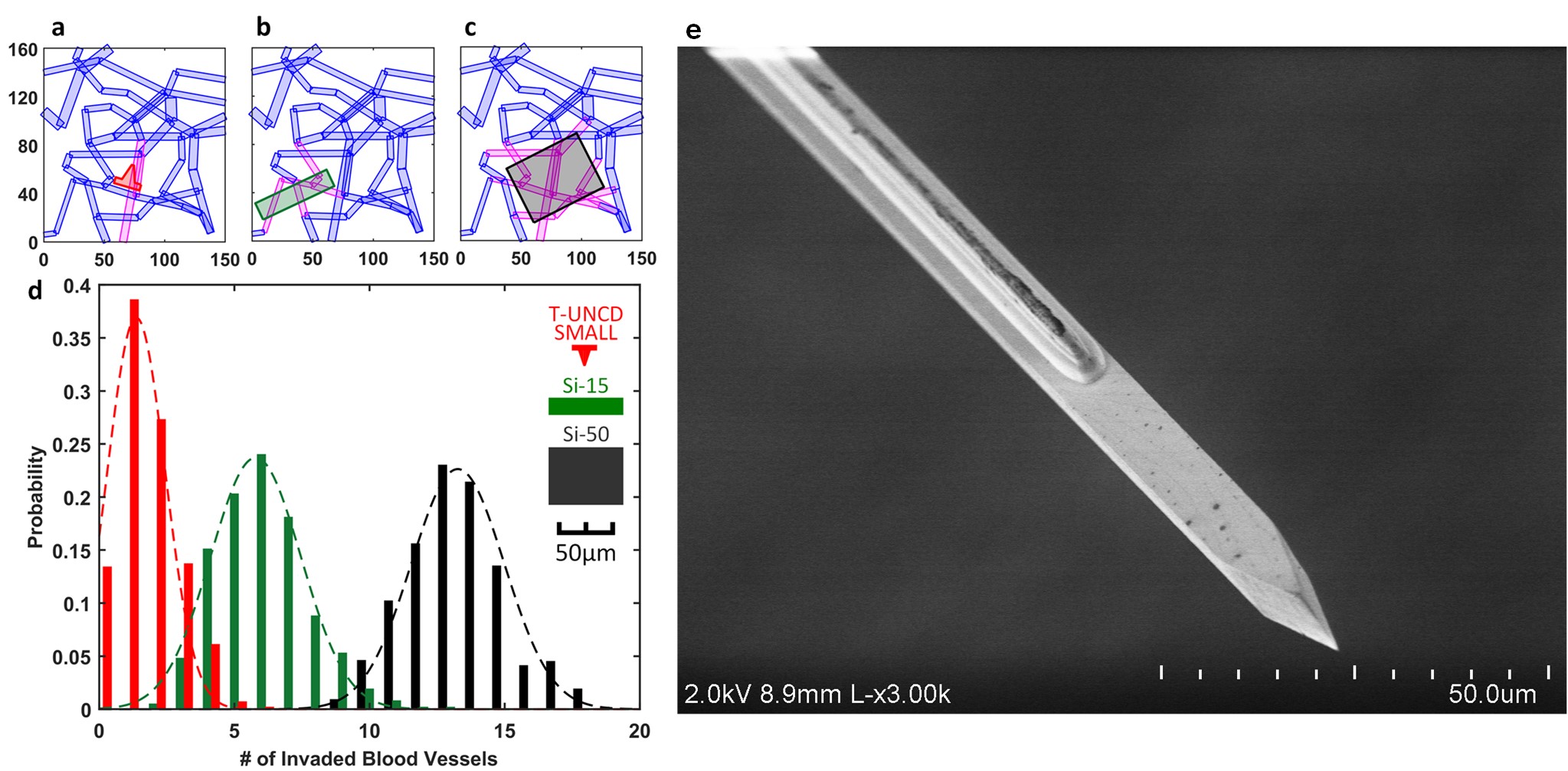


Supplementary Figure 1 | Potential for ultra-fine UNCD devices. a-d, Simulation of blood vessel invasion based on various probe cross-sections. Examples of invasion analysis with a, T shape with 22 x 5 µm on the planar portion and 13.5µm-deep, 11 to 2µm-wide trapezoid for the vertical support (red, T-UNCD SMALL). b, 65 µm by 15 µm rectangle (green, Si-15). c, 65 µm by 50 µm rectangle (black, Si-50). d, Simulation of 1,000 insertions result in a Gaussian distribution of the numbers of invaded blood vessels. Mean values of three geometries from (a-c) were 1.4, 5.8, and 13.3, respectively. Invaded blood vessels in (a-c) indicated in magenta. X and Y labels in (a-c) indicated the coordinates in µm. e, Demonstration that ultra-fine UNCD probes can be manufactured with good yield -- this diamond shuttle is much smaller than even T-UNCD-SMALL, at 8-µm wide.

## Supplemental Note 2: Limits to conformal trench filling with UNCD

The sticking coefficient (η_0_) is a property of CVD deposition and is the most important parameter in determining a film’s conformation around 3-D structures. η_0_ is dependent on pressure, temperature, and the nature of each species, etc. Theoretically, the shape of the trench and the sticking coefficient determine the deposition profile. As mentioned in the Results section, the sticking coefficient of UNCD deposition varies in a large range and was unknown for the system we used at the time of deposition. The challenge of trench filling of UNCD is the moderate conformality of the UNCD film. Our model estimated that γ= 0.079, which is a moderate sticking coefficient^4^. We resolved the issue by creating a tapered trench (lower aspect ratio) but the curved shape of the T-beam also minimizes high-stress points that would exist around a sharp T-beam geometry. Furthermore, characterizing the critical factors contributing to the sticking coefficient is a long experimental process best done when the process is done in-house as opposed to a commercial service.

Ganguli et al^5^ and Komiyama et al^6^ suggested the governing equation of deposition model based on the mass conservation and it was reformulated as shown in (2). It was assumed that the width of trench is constant along with the depth as well as time. The assumptions are inappropriate due to tapered profile and increasing film thickness over time. The derivative equation becomes unsolvable form and analytical solution cannot be derived.

$\frac{d}{dy}\left(\frac{2W^{2}}{3\eta_{0}}\frac{dC}{dy}\right)=C$ (2)

where W is the width of trench, η_0_ is the sticking coeffieient, and C is the concentration of reactant.

Instead, we converted the derivative equation to a discrete form and iterated the calculation according to the depth and the time. The film thickness deposited on discrete coordinates of y during a unit time was calculated with the fixed width and then the width was updated considering the deposited film for the following calculation during the next unit time. The calculation was iterated until the time when the targeted total thickness was achieved.

The model predicted a slight hollow in the middle of the trench since the etched profile was not linear and the sidewall angle of some portion was above the critical angle (70ˇ) for void-free filling even though the averaged angle was more tapered. Since a small void has a small change in the moment of inertia, we also expect a small loss of mechanical strength.

Finally, it is noted we limited our film thickness to 11 µm because the manufacturer warned that the residual stress of UNCD film is not consistent during deposition and the resulting gradient make it challenging to control or compensate for residual stress with a thick film UNCD. We consistently found that at this thickness, and within our tested length of 6.5 mm, there was no measurable out-of-plane bending. These results were very consistent. The moderate sticking coefficient and stress gradient will eventually limit the aspect ratio and film thickness so the upper limit of strength we estimate to be in the range of 640 mN based on our current film properties. This is sufficient for all of the animal models for which we currently have data for.


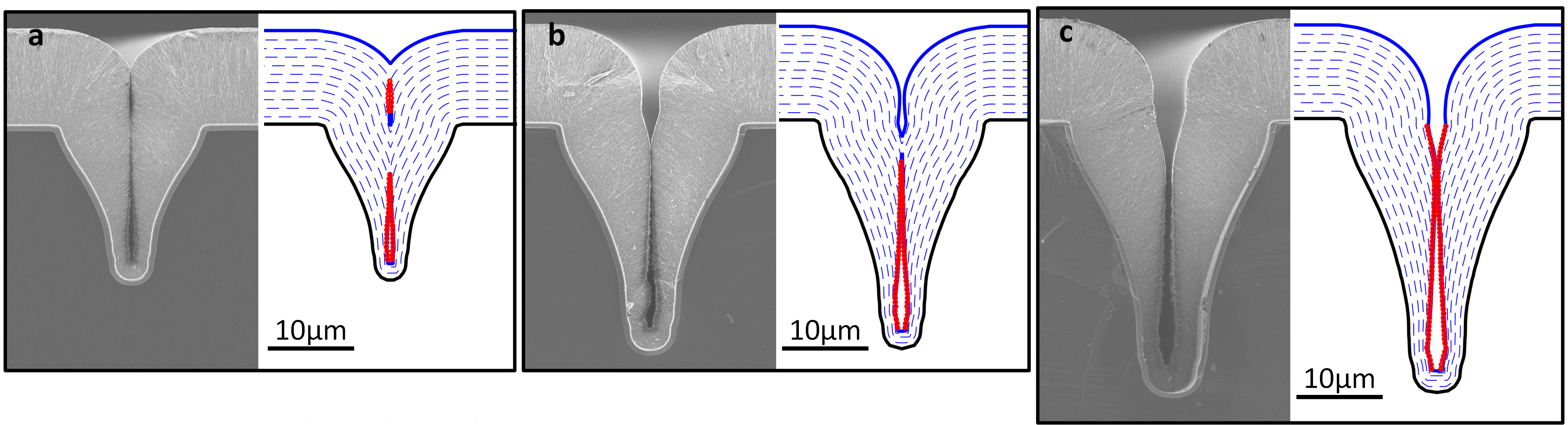


Supplementary Figure 2 | Simulation result of UNCD profile modeling. a, design A with 18.3µm-deep trench, R^2^=0.957. b, design B with 26.9µm-deep trench, R^2^=0.946. c, design C with 32.2µm-deep trench, R^2^=0.943. Blue solid line is the final outline of UNCD film. Blue dashed lines show the progression of deposition. Red lines indicate the measured profiles of UNCD film.

**
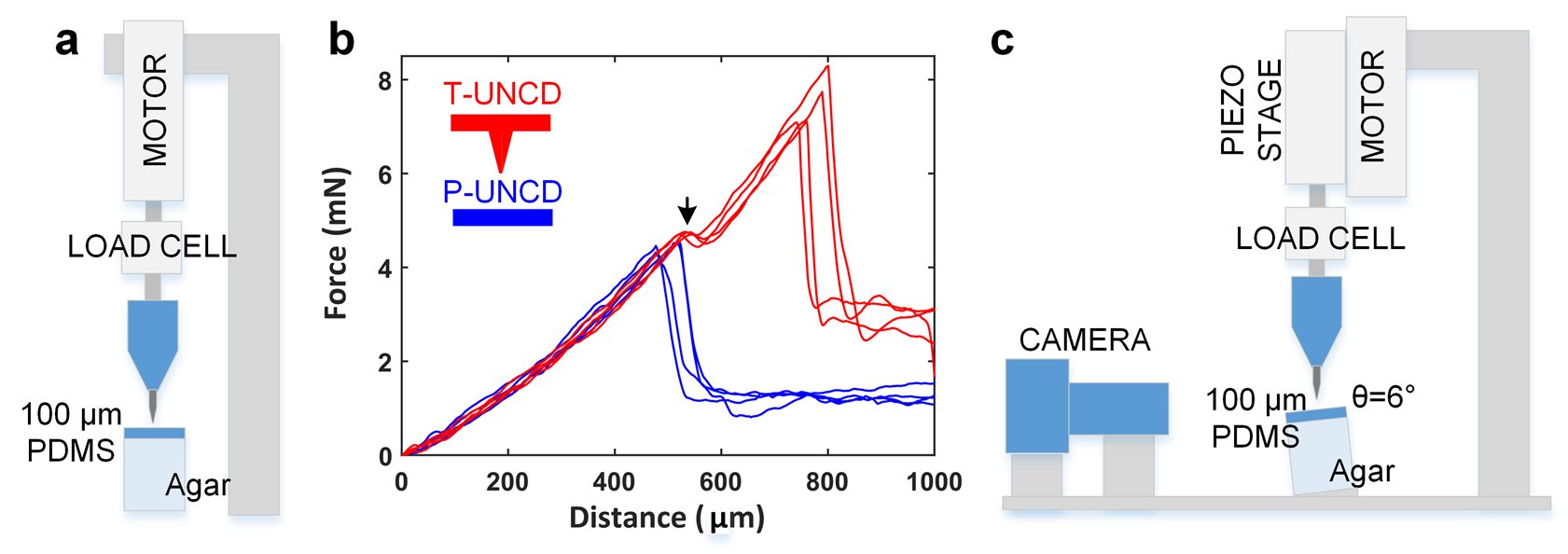
**

**Supplementary Figure 3 | Measurement setup and force-distance curve for insertion through phantom. a,** Measurement setup with a motor, a load cell and a tissue phantom with 100µm-thick PDMS. The phantom uses 100-µm thick layer of Sylgard 184 silicone (1-2 MPa^7^) over 0.7% agar to represent a membrane and soft nervous tissue. **b,** Force-distance curve for P- UNCD and T-UNCD. T-UNCD showed two peaks (arrow on red line), with the earlier one indicating the insertion of the planar tip. Insertion speed 0.01mm/s and acceleration 0.1mm/s^2^. **c**, Measurement setup that includes a camera with 4X macro lens to measure maximum compression distance. Piezomotor from PiezoConcept, Inc designed for high-precision microscope stages was used here to enable uni-axial sinusoidal oscillations of up to 70-µm peak-to-peak.

**
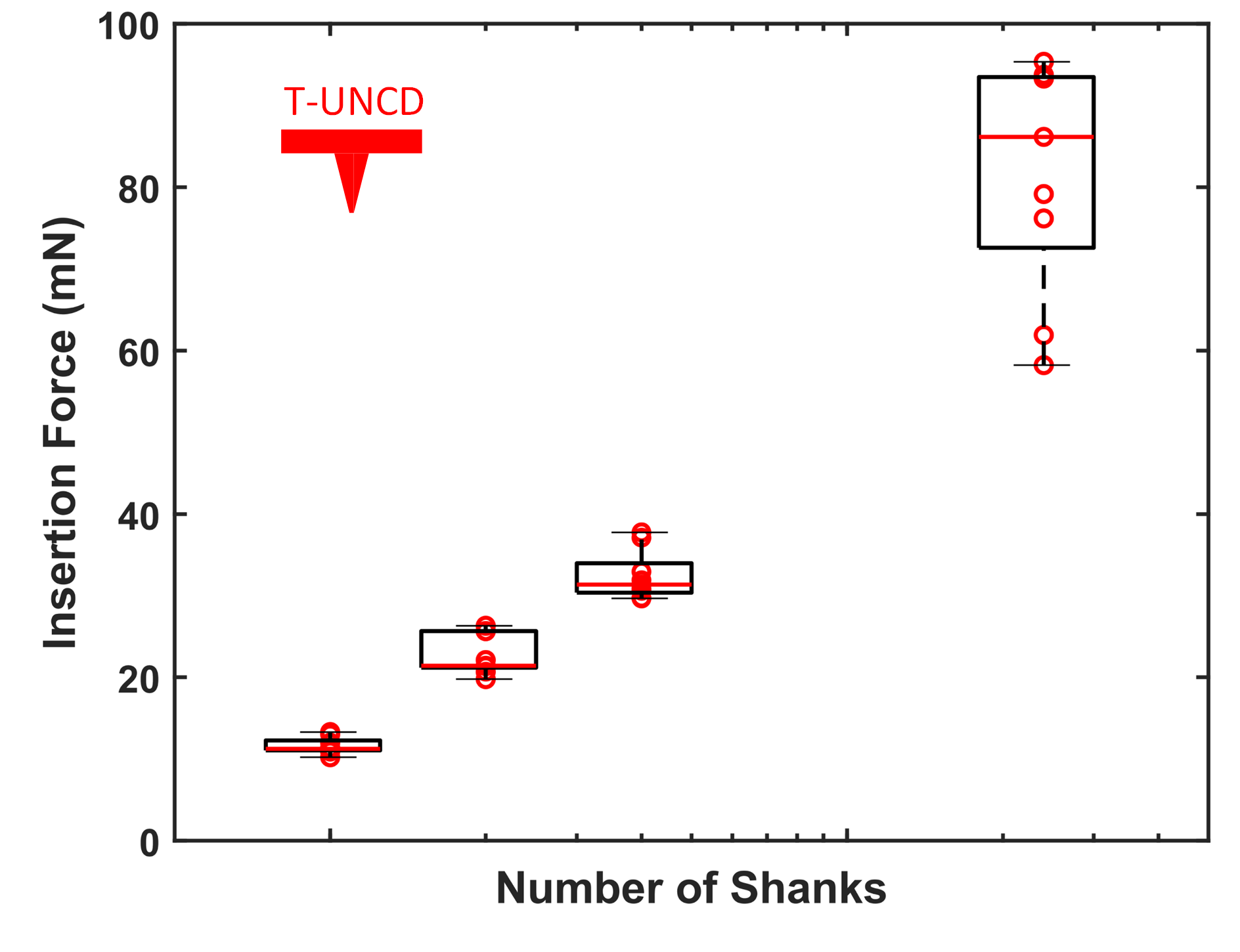
**

**Supplementary Figure 4 | Phantom insertion force of 1,2,4 and 24 shanks for UNCD.** To achieve 24 shanks, we arranged six 4-shank arrays in a hexagonal pattern. The force per shank in this case is 3.4 mN, a 66% and 72% force reduction relative to the 4-shank and 1-shank designs (Figure 3). This mechanism of local force reduction is yet unknown and may vary from shank to shank so more research on the local stress may help to reduce the size of a large number of shanks relative to the single shank version.

**
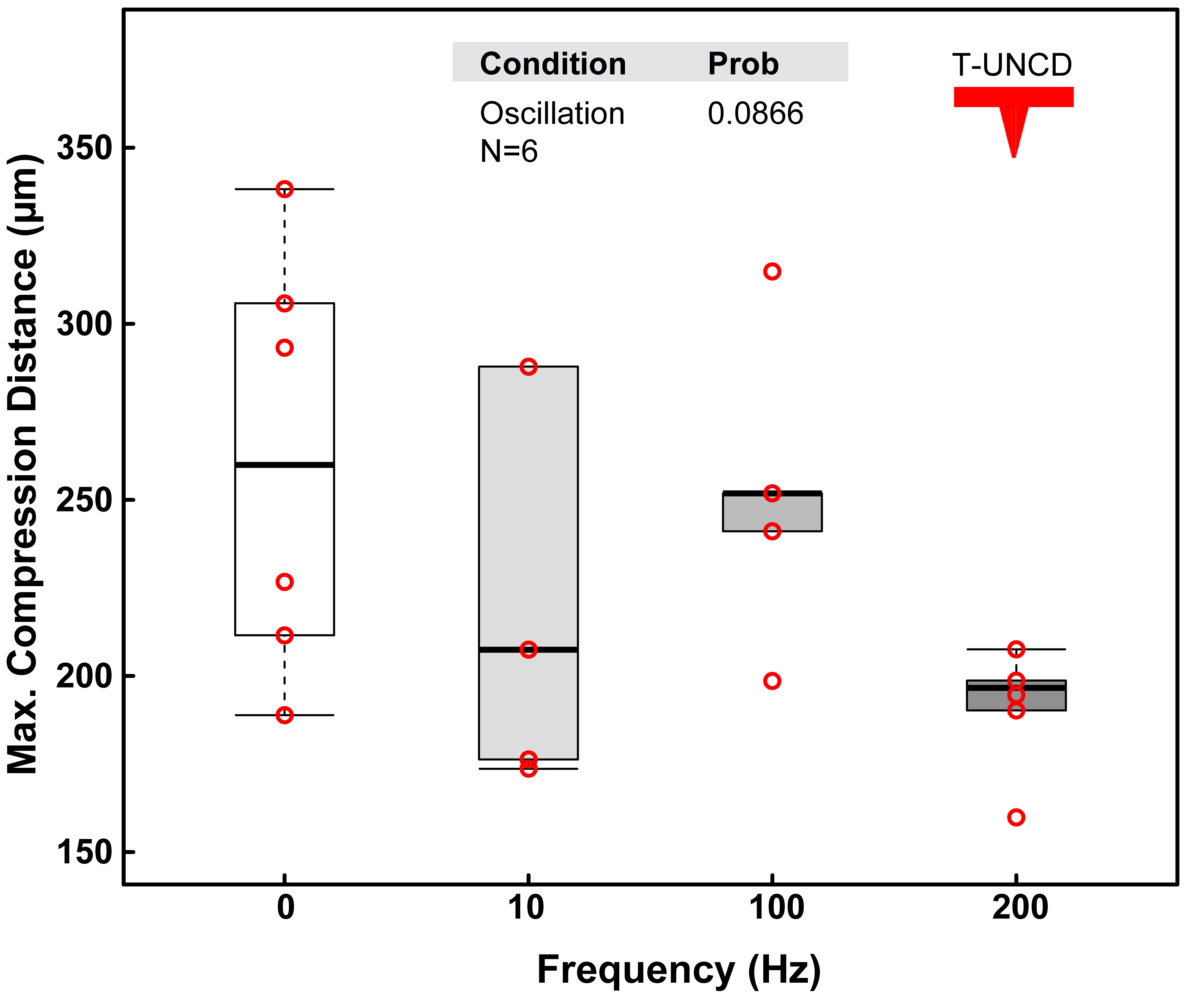
**

**Supplementary Figure 5 | Maximum compression distance of UNCD shuttle with varying oscillation frequency at 2 mm/s in tissue phantom.**

**
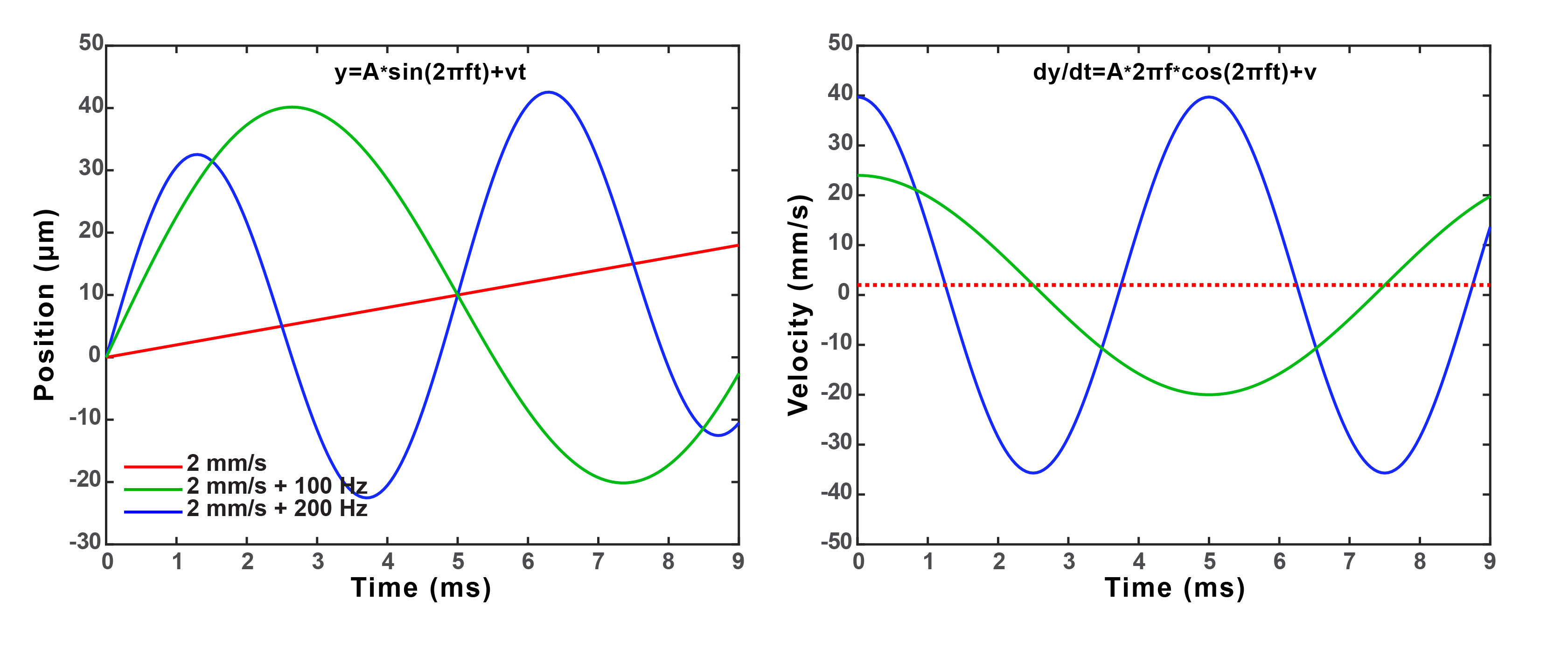
**

**Supplementary Figure 6 | Kinematics of instantaneous insertion speed using a piezostage motor with uniaxial motion.** The position equation and its first derivative are defined in the figures where A is maximum amplitude for a given oscillation. Beyond 200 Hz, the amplitude of this piezostage, HS1.70 from PiezoConcept, was dampened (see Supp. Table 1).

**Supplementary Table 1 | Peak Velocity and Acceleration of All Phantom Conditions Tested**

|  | **Frequency** | | | |
| --- | --- | --- | --- | --- |
|  | **10** | **100** | **200** | **400^A^** |
| Max amplitude (µm) | 35 | 35 | 30 | 13.6 |
| Max speed (mm/s) | 2.2  + linear sp | 22  + linear sp | 38  + linear sp | 34  + linear sp |
| Max acceleration (m/s^2^) | 0.1 | 14 | 47 | 86 |

Note A: 400 Hz was only tested at 0.01 mm/s and only for 2 trials. Given it was a slower speed than 200 Hz and showed no improvement in those 2 trials we dropped this condition from the study.

**Supplementary Figure 7 | Oscillation and force testing of a 2-µm tip pipette at low speeds in tissue phantom.** Glass pipettes are still a common implantable sensor for neuroscience and makes a useful model understanding the effect of device cross-sectional area, which is monotonically increasing. **a,** Oscillation significantly reduces the maximum compression distance (p=0.00074, N>6). **b,** While the tip is very sharp, we observe that the larger cross-sectional area dominates the force response, as reported by others^8^.

**
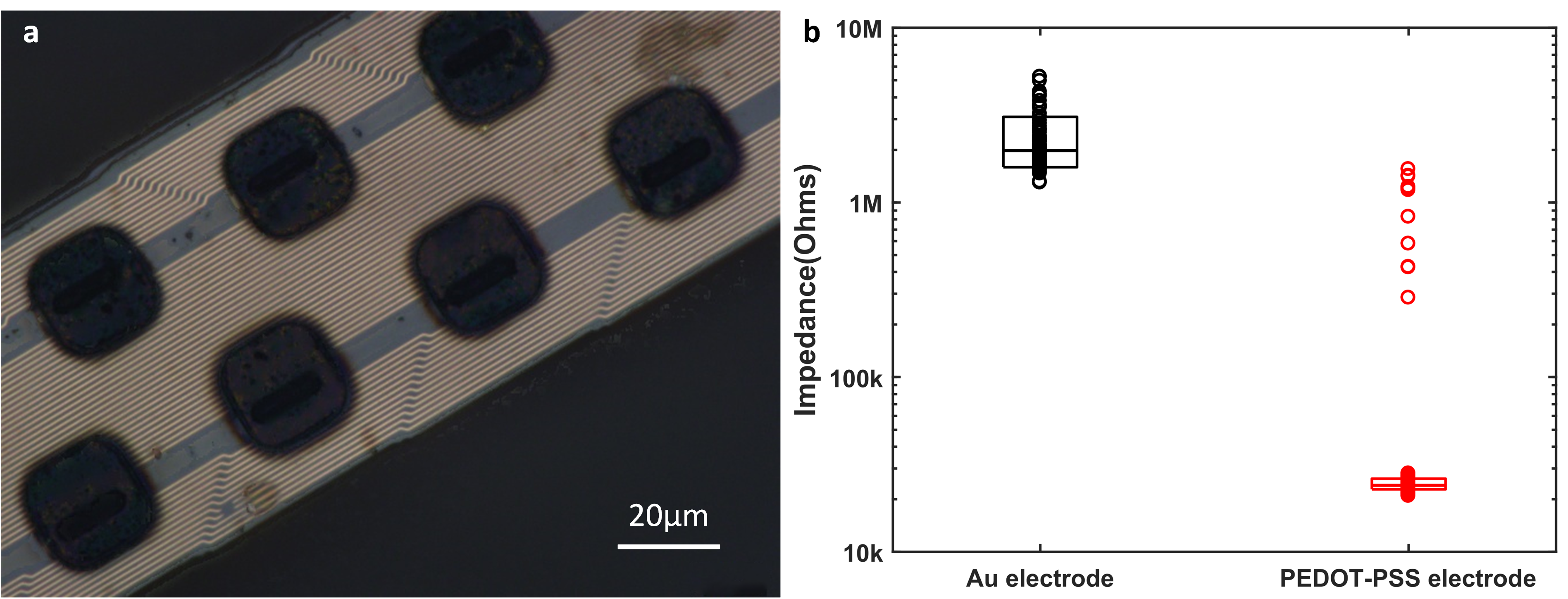
**

**Supplementary Figure 8 | Picture and impedance of PEDOT:pTS coated electrodes. a,** Poly(3,4-ethylenedioxythiophene: Sodium p-toluenesulfonate was plated on Au electrodes with a current of 690 nA and duration of 10 minutes as described in Patel, et. al 2016^9^. **b,** Impedances at 1kHz for non-coated Au electrodes and PEDOT-pTS coated electrodes, N=120. Top, middle and bottom lines of box plots indicates 90% value, harmonic mean, and 10% value, respectively. For the PEDOT-pTS coated electrodes, 14 electrodes with relatively high impedance were excluded as outliers.

## Supplementary Note 3: Detailed results of feline in-vivo experiments

This note includes additional descriptions of the electrode performance. In the first animal experiment a total of 28 different channels had a unit out of 42 functional channels during six sensory input trials. The per trial average was 11 units across 8 channels with single unit amplitudes between 35-323 µV peak-to-peak (mean 68 µVp-p, median 47 µVp-p).

In the second animal experiment an average of 78 units across 32 channels were observed during each of eight evoked trials during testing with the electrode in a fixed position, with single unit amplitudes between 39-1313 µV peak-to-peak (mean 123 µVp-p, median 80 µVp-p). Across multiple trials in this fixed position, a total of 48 different channels had a unit (167 non-unique units observed) out of 59 functional channels, spanning 1.06 mm in depth. When this same probe was retracted, units clearly moved between electrodes, including appearing on electrodes that were deeper in the tissue, suggesting that the tip of the array may have initially been in an inactive region of the DRG or in the ventral root (Fig. 7a). During movement of the probe, a total of 249 units were identified, with all 59 functional channels having activity across the full 1.2 mm span. Single units during the probe movement trial were within 50-1273 µV peak-to-peak (mean 199 µVp-p, median 125 µVp-p).

As the channels within the electrode array were separated by 40 µm, some units appear on multiple channels and we used careful manual sorting followed by comparison of waveform shapes across neighboring channels to report putative units. High-density arrays, like that used here, are best employed using an unbiased polytrode analysis as others have demonstrated^10–12^ to eliminate double counts but also to increase the number of unique identifiable units when performing an in-depth mapping study^13^.

**
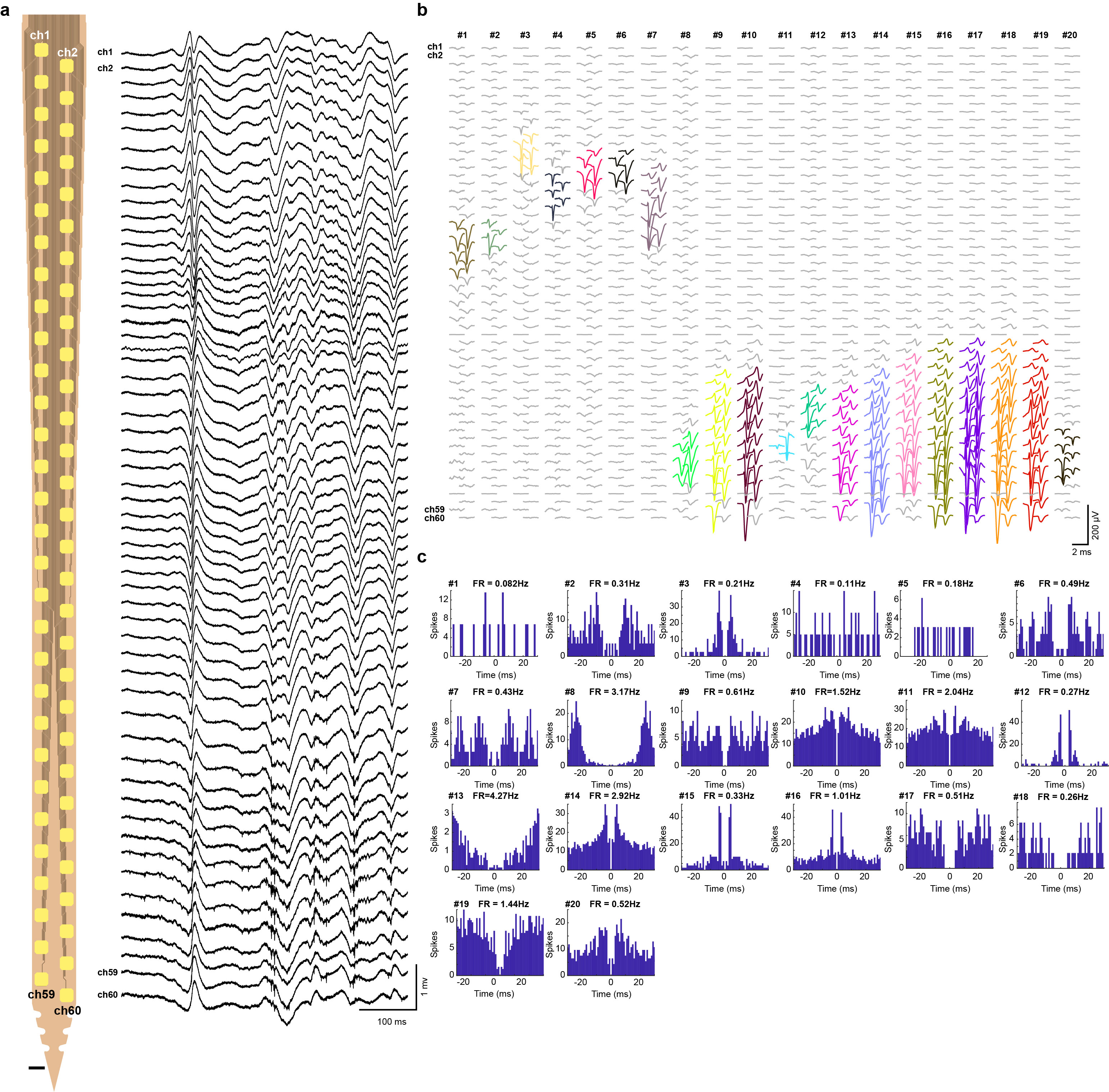
**

**Supplementary Figure 9 | Recorded neural signal from somatosensory cortex of rat. a,** Wide-band (0.3 – 3000 Hz) spiking activity recorded on a flexible electrode from S1FL region of an anesthetized rat (right) and a schematic of the probe sites (left). Note the spiking activity at the bottom of the shank. Scale = 20 µm. **b,** Recording of high number of well-isolated single units. The spiking data was quantified for 20 putative single units recorded simultaneously from S1FL (same animal and session as in **a**). Plots show average single-unit triggered waveforms (n = number of spikes, at least 500) on all channels. Note the single unit activity is present across multiple channels but the larger than expected range is due to the movement of probe relative to the brain tissue over the course of 30 minutes. **c,** Auto-correlation histograms of the 20 putative single units (same as **b**). Mean firing rate (FR) value is shown for each unit above the histogram.

**Supplementary Note 4: Calculation of moment of inertia of trapezoid shaped T-beam**

The moment of inertia of trapezoid shaped T-beam was calculated by addition of moment of inertia of each part, rectangular base part and vertical part which is trapezoid shaped. In the calculation of the axis of the width, x-axis, the centroid of each part and the moment of inertia of each part about its centroid were calculated. Then, the moment of inertia of each part was converted to that about the centroid of the whole structure and added. In the calculation of the axis of the height, y-axis, the moment of inertia of each part was simply added since the centroids of the base part, the vertical part and the whole structure are in the same about y-axis.

$$\begin{matrix} A_{1}=Wh_{1},\text{ }A_{2}=\frac{a+b}{2}h_{2} \\ Y_{C1}=\frac{h_{1}}{2}+h_{2},\text{ }Y_{C2}=\frac{h_{2}}{3}\frac{2a+b}{a+b},\text{ Y}_{\text{c}}=\frac{A_{1}Y_{C1}+A_{2}Y_{C2}}{A_{1}+A_{2}} \\ I_{x}=\frac{Wh_{1}^{3}}{12}+A_{1}(Y_{C1}-Y_{C})^{2}+\frac{h_{2}^{3}}{36}\frac{a^{2}+4ab+b^{2}}{a+b}+A_{2}(Y_{C2}-Y_{C})^{2} \\ I_{y}=\frac{W^{3}h_{1}}{12}+\frac{h_{2}}{36}\left(a+b\right)\left(a^{2}+b^{2}\right) \end{matrix}$$

Where W is the width of the base part, h_1_ is the thickness of the base part, a is the top width of the trapezoid of the vertical part, b is the bottom width of the trapezoid of the vertical part, h_2_ is the height of the vertical part, A_1_ is the area of the base part, A_2_ is the area of the vertical part, Y_C1_ is the centroid of the base part, Y_C2_ is the centroid of the vertical part, Y_C_ is the centroid of the whole structure, I_x_ is the moment of inertia along x-axis, the axis of the width and I_y_ is the moment of inertia along y-axis, the axis of the height.

**Supplementary Note 4: Calculation of moment of inertia of trapezoid shaped T-beam**


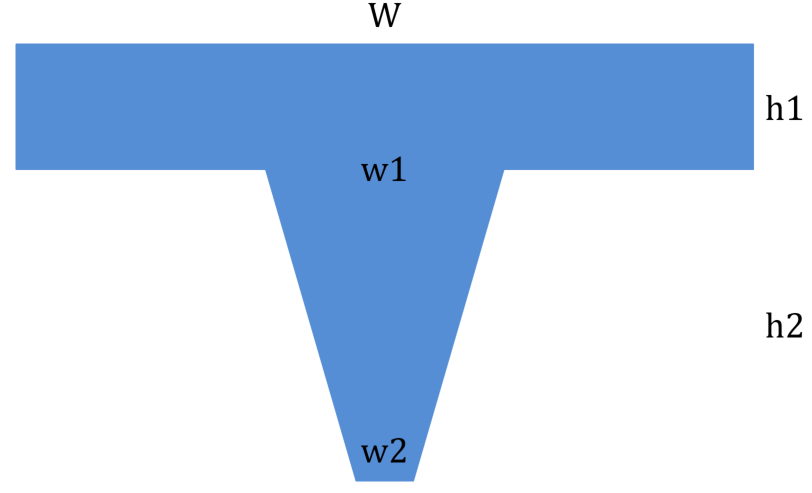


The moment of inertia of trapezoid shaped T-beam was calculated by addition of moment of inertia of each part, rectangular base part and vertical part which is trapezoid shaped. In the calculation of the axis of the width, x-axis, the centroid of each part and the moment of inertia of each part about its centroid were calculated. Then, the moment of inertia of each part was converted to that about the centroid of the whole structure and added. In the calculation of the axis of the height, y-axis, the moment of inertia of each part was simply added since the centroids of the base part, the vertical part and the whole structure are in the same about y-axis.

$$\begin{matrix} A_{1}=Wh_{1},\text{ }A_{2}=\frac{a+b}{2}h_{2} \\ Y_{C1}=\frac{h_{1}}{2}+h_{2},\text{ }Y_{C2}=\frac{h_{2}}{3}\frac{2a+b}{a+b},\text{ Y}_{\text{c}}=\frac{A_{1}Y_{C1}+A_{2}Y_{C2}}{A_{1}+A_{2}} \\ I_{x}=\frac{Wh_{1}^{3}}{12}+A_{1}(Y_{C1}-Y_{C})^{2}+\frac{h_{2}^{3}}{36}\frac{a^{2}+4ab+b^{2}}{a+b}+A_{2}(Y_{C2}-Y_{C})^{2} \\ I_{y}=\frac{W^{3}h_{1}}{12}+\frac{h_{2}}{36}\left(a+b\right)\left(a^{2}+b^{2}\right) \end{matrix}$$

Where W is the width of the base part, h_1_ is the thickness of the base part, a is the top width of the trapezoid of the vertical part, b is the bottom width of the trapezoid of the vertical part, h_2_ is the height of the vertical part, A_1_ is the area of the base part, A_2_ is the area of the vertical part, Y_C1_ is the centroid of the base part, Y_C2_ is the centroid of the vertical part, Y_C_ is the centroid of the whole structure, I_x_ is the moment of inertia along x-axis, the axis of the width and I_y_ is the moment of inertia along y-axis, the axis of the height.

**Supplementary Video 1 | Side-by-side comparison of UNCD insertion without and with 200 Hz oscillation.** The linear speed of insertion was 0.01mm/s for both. Link for video will be provided in a peer-reviewed journal article.

**Supplementary Video 2 | In vivo insertion through rat dura and retraction leaving flexible array implanted.** The polyimide electrode array on T-UNCD shuttle was implanted into the motor cortex and the T-UNCD shuttle was successfully retracted, leaving the polyimide array inside. The insertion was conducted at 0.01mm/s speed with 200Hz oscillation. Retraction occurred in steps of 50 to 100 µm to ensure separation was visible before larger movements were made. Link for video will be provided in a peer-reviewed journal article.

**Supplementary Video 3 | Example of insertion failure due to tissue movement during *ex-vivo* trial.** The insertion speed was 0.01mm/sec and the travel distance was 1.5mm. The video showed the buckling failure of P-UNCD during the insertion trial into DRG L7 explanted from a feline. Link for video will be provided in a peer-reviewed journal article.

## References:

1. Gilletti, A.; Muthuswamy, J. Brain Micromotion around Implants in the Rodent Somatosensory Cortex. *Journal of neural engineering* **2006**, *3*, 189.

2. Boehler, C.; Kleber, C.; Martini, N.; Xie, Y.; Dryg, I.; Stieglitz, T.; Hofmann, U. G.; Asplund, M. Actively Controlled Release of Dexamethasone from Neural Microelectrodes in a Chronic in Vivo Study. *Biomaterials* **2017**.

3. Luan, L.; Wei, X.; Zhao, Z.; Siegel, J. J.; Potnis, O.; Tuppen, C. A.; Lin, S.; Kazmi, S.; Fowler, R. A.; Holloway, S.; Dunn, A. K.; Chitwood, R. A.; Xie, C. Ultraflexible Nanoelectronic Probes Form Reliable, Glial Scar–free Neural Integration. *Science Advances* **2017**.

4. Eckert, M.; Neyts, E.; Bogaerts, A. Molecular Dynamics Simulations of the Sticking and Etch Behavior of Various Growth Species of (Ultra)Nanocrystalline Diamond Films. *Chemical Vapor Deposition* **2008**.

5. Ganguli, S.; Costello, S. P.; Gill, W. N. Theory of Free Boundary Step Coverage in Chemical Vapor Deposition. *Ind. Eng. Chem. Res* **1996**, *34*, 3380–3391.

6. Komiyama, H.; Shimogaki, Y.; Egashira, Y. Chemical Reaction Engineering in the Design of CVD Reactors. *Chemical Engineering Science* **1999**, *54*, 1941–1957.

7. Johnston, I. D.; McCluskey, D. K.; Tan, C. K. L.; Tracey, M. C. Mechanical Characterization of Bulk Sylgard 184 for Microfluidics and Microengineering. *Journal of Micromechanics and Microengineering* **2014**, *24*.

8. Römgens, A. M.; Bader, D. L.; Bouwstra, J. A.; Baaijens, F. P. T.; Oomens, C. W. J. Monitoring the Penetration Process of Single Microneedles with Varying Tip Diameters. *Journal of the Mechanical Behavior of Biomedical Materials* **2014**, *40*, 397–405.

9. Patel, P. R.; Zhang, H.; Robbins, M. T.; Nofar, J. B.; Marshall, S. P.; Kobylarek, M. J.; Kozai, T. D. Y.; Kotov, N. A.; Chestek, C. A. Chronic in Vivo Stability Assessment of Carbon Fiber Microelectrode Arrays. *Journal of Neural Engineering* **2016**, *13*, 66002.

10. Rossant, C.; Kadir, S.; Harris, K. Spike Sorting for Large Dense Electrode Arrays: User Interface Software. *Annual Meeting of the Society for Neuroscience* **2013**.

11. Chung, J. E.; Magland, J. F.; Barnett, A. H.; Tolosa, V. M.; Tooker, A. C.; Lee, K. Y.; Shah, K. G.; Felix, S. H.; Frank, L. M.; Greengard, L. F. A Fully Automated Approach to Spike Sorting. *Neuron* **2017**, *95*, 1381–1394.e6.

12. Jun, J. J.; Mitelut, C.; Lai, C.; Gratiy, S. L.; Anastassiou, C. A.; Harris, T. D. Real-Time Spike Sorting Platform for High-Density Extracellular Probes with Ground-Truth Validation and Drift Correction. *bioRxiv* **2017**, 1–29.

13. Gray, C. M.; Maldonado, P. E.; Wilson, M.; McNaughton, B. Tetrodes Markedly Improve the Reliability and Yield of Multiple Single-Unit Isolation from Multi-Unit Recordings in Cat Striate Cortex. *Journal of neuroscience methods* **1995**, *63*, 43–54.
